# Diversity of Expression Patterns of *Lr34*, *Lr67*, and Candidate Genes towards *Lr46* with Analysis of Associated miRNAs in Common Wheat Hybrids in Response to *Puccinia triticina* Fungus

**DOI:** 10.3390/cimb46060329

**Published:** 2024-05-31

**Authors:** Julia Spychała, Agnieszka Tomkowiak, Aleksandra Noweiska, Roksana Bobrowska, Jan Bocianowski, Aleksandra Sobiech, Michał Tomasz Kwiatek

**Affiliations:** 1 Department of Genetics and Plant Breeding, Faculty of Agronomy, Horticulture and Biotechnology, Poznań University of Life Sciences, Dojazd 11, 60-632 Poznań, Poland; julia.spychala@up.poznan.pl (J.S.); aleksandra.noweiska@up.poznan.pl (A.N.); roksana.bobrowska@up.poznan.pl (R.B.); aleksandra.sobiech@up.poznan.pl (A.S.); m.kwiatek@ihar.edu.pl (M.T.K.); 2 Department of Mathematical and Statistical Methods, Poznań University of Life Sciences, 60-637 Poznań, Poland; 3 Plant Breeding and Acclimatization Institute—National Research Institute in Radzików, 05-870 Błonie, Poland

**Keywords:** leaf rust, hybrid breeding, APR resistance, slow rusting, candidate genes

## Abstract

Leaf rust caused by *Puccinia triticina* (Pt) is one of the most dangerous diseases causing significant losses in common wheat crops. In adult plants resistant to rust, a horizontal adult plant resistance (APR) type is observed, which protects the plant against multiple pathogen races and is distinguished by greater persistence under production conditions. Crucial pleiotropic slow-rust genes such as *Lr34*, *Lr46*, *Lr67*, and *Lr68*, in combination with other genes of lesser influence, continue to increase durable resistance to rust diseases. Based on our previous results, we selected four candidate genes for *Lr46* out of ten candidates and analysed them for expression before and after inoculation by *P. triticina*. As part of our study, we also investigated the expression patterns of miRNA molecules complementary to *Lr34* and the candidate genes. The aim of the study was to analyse the expression profiles of candidate genes for the *Lr46* gene and the *Lr34* and *Lr67* genes responsible for the differential leaf-rust resistance of hybrid forms of the F1 generation resulting from crosses between the Glenlea cultivar and cultivars from Polish breeding companies. In addition, the expression of five miRNAs (tae-miR9653b, tae-miR5384-3p, tae-miR9780, tae-miR9775 and tae-miR164), complementary to *Lr34*, and selected candidate genes were analysed using stem-loop RT-PCR and ddPCR. Biotic stress was induced in adult plants by inoculation with *Pt* fungal spores, under controlled conditions. Plant material was collected before and 6, 12, 24, and 48 h after inoculation (hpi). Differences in expression patterns of *Lr34*, *Lr67*, and candidate genes (for *Lr46*) were analysed by qRT-PCR and showed that gene expression changed at the analysed time points. Identification of molecular markers coupled to the *Lr* genes studied was also carried out to confirm the presence of these genes in wheat hybrids. qRT-PCR was used to examine the expression levels of the resistance genes. The highest expression of *Lr46/Yr29* genes (*Lr46-Glu2*, *Lr46-RLK1*, *Lr46-RLK2*, and *Lr46-RLK3*) occurred at 12 and 24 hpi, and such expression profiles were obtained for only one candidate gene among the four genes analysed (*Lr46-Glu2*), indicating that it may be involved in resistance mechanisms of response to *Pt* infection.

## 1. Introduction

Common wheat (*Triticum aestivum* L.) is one of the paramount crop species of enormous importance. Worldwide, wheat cultivation is extremely relevant which is connected with its large share in human and animal nutrition and high yield potential. Currently, the primary directions of wheat breeding focus mainly on high yield, high commercial quality, and resistance to biotic and abiotic stresses [1]. Leaf rust outbreaks can occur with high frequency causing yield losses of up to 70% [2,3]. In addition, leaf rust causes a reduction in grain quality and the risk of mycotoxin contamination. The increase in rust infections is linked to the continuous diversification of naturally occurring rust populations, leading to the emergence of increasingly resistant pathotypes [2,4]. The most effective and environmentally friendly method of controlling fungal diseases found in wheat crops is to grow resistant varieties. However, producing a variety with the right combinations of resistance genes requires an understanding of genetic variation and *Pt* composition [5].

Plant crops are susceptible to diseases caused by fungal and bacterial pathogens, nematodes, and viruses. The economic value of the species drives investment in research into these diseases. Crop improvement programmes have always focused on disease resistance. Plant breeders focus on implementing diverse sources of resistance to achieve sustainable disease control which is hampered by ever-evolving pathogen populations creating new virulent races. Therefore, knowledge of genetic variation in resistance, the evolutionary capacity of pathogens, and the selection of modern breeding methods are essential for successful resistance breeding. Currently, wheat breeders are focusing on using resistance genes that are non-race-specific to the fungus. Breed-unspecific resistance to leaf rust is characterized by a partial resistance phenotype activated in plants at the adult stage. To ensure sustainable rust control, new sources of resistance must be found and identified [4,6]. Genetically determined resistance to *Puccinia triticina* (*Pt*) Eriks has been characterised in both young plants (seedling resistance; SR) and plants at the mature stage (adult plant resistance; APR). At the seedling stage, SR is controlled by the R genes. In wider use, most of the R genes that provide race-specific Pt resistance are only effective for a few years [3,7]. Adult plant resistance (APR) sources provide low to moderate levels of resistance at stages after sowing. This is referred to as the slow-rusting effect. Achieving resistance that is commercially acceptable requires a combination of more than two APR genes [7].

Among the most threatening diseases leading to significant losses in the world’s wheat crops is leaf rust caused by the fungus *Puccinia triticina* (*Pt*) Eriks. It occurs more frequently than other rust diseases and is more prevalent worldwide [8]. All known genes for resistance to leaf rust and stripe rust, also known as yellow rust, are classified according to a specific nomenclature that assigns the symbol *Lr* (Leaf rust) and *Yr* (Yellow rust) to such genes. Over the past few decades, numerous research work has led to the mapping of *Lr* genes and many important quantitative trait loci (QTLs) on different chromosomes, which have been identified using DNA markers [9,10]. Leaf-rust resistance genes, designated *Lr1* to *Lr81*, have been described, as well as several QTLs associated with resistance [11]. However, most of the identified *Lr* genes are no longer used in breeding because they are not effective against recent *Pt* races [11,12]. Strategic breeding of cultivars with resistance genes through pyramiding can be an effective aid in reducing the chances of virulent breeds evolving.

Nowadays, important slow-rust pleiotropic genes such as *Lr34*, *Lr46*, *Lr67*, and *Lr68* continue to improve partial, long-term resistance [13]. Partial resistance is due to the slow-rust phenotype, which has a longer latent period, resulting in the pathogen developing more slowly [14]. The *Lr34*, *Lr42*, *Lr46*, *Lr67*, and *Lr68* genes have been identified as non-racial APR genes in hexaploid wheat. The *Lr34* encodes an ABC (ATP binding cassette) transporter from the ABCG subfamily, while the *Lr67* gene encodes a hexose transporter with a similar affinity to glucose. The susceptible and resistant *Lr67* allele differ by two amino acid residues [15,16]. In addition, these genes are not only structurally different from known R genes, but encode proteins of different classes [17]. The *Lr34res* gene is one of the most durable sources of quantitative fungal resistance in wheat. It has been shown to be effective against powdery mildew and spot blotch as well as leaf rust, stem rust, and stripe rust [18]. The leaf-rust resistance genes *Lr34* and *Lr46* act on all known races of *P. triticina* and are present in many of the wheat cultivars currently grown. Both genes regulate an intermediate level of leaf-rust resistance, which is most pronounced at the adult plant stage and is associated with persistent leaf-rust resistance. The *Lr67* gene, like the *Lr68*, *Lr74*, *Lr75*, and *Lr77* genes, has the potential to provide resistance over a long period of time. However, a single *Lr67* gene does not provide high resistance, but wheat cultivars with a combination of adult plant resistance genes or seedling resistance genes with adult plant genes can be highly resistant [19].

The *Lr46* gene was first described in the cultivar ‘Pavon 76’ [20]. The *Lr46* confers broad-spectrum resistance to four biotrophic fungal pathogens: *P. triticina* (*Lr46*), *P. striiformis* (*Yr29*), *P. graminis* f. sp. *tritici* (*Sr58*), and *Blumeria graminis* f. sp. *tritici* (*Pm39*) [12,21]. A locus *Lr46*/*Yr29*/*Sr58*/*Pm39* on chromosome 1BL is associated with resistance to multiple pathogens, but the specific gene has still not been identified. A number of candidate genes for *Lr46*/*Yr29* were selected and analysed [22]. However, it is still unclear whether this locus of resistance to multiple pathogens is the result of the pleiotropic action of a single gene or multiple related genes. Moreover, this gene belongs to a group of genes that show an effect known as “slow rusting”, i.e., partial resistance during pathogen infection.

We analyse the expression profiles for nine of the ten candidate genes included in the *Lr46/Yr29* locus at five time points (0, 6, 12, 24, and 48 h post-inoculation (hpi)): TraesCS1B02G453900.1, TraesCS1B02G454200.1, TraesCS1B02G454500.1, TraesCS1B02G454000.2, TraesCS1B02G454100.1, TraesCS1B02G454400.1, TraesCS1B02G454600.1, TraesCS1B02G453700.1 and TraesCS1B02G455000.1 [22]. In a study by Cobo et al. [22], they mapped the *QYr.ucw-1BL* region on chromosome 1BL, which coincided with the current resistance maps of the *Pt Yr29* resistance gene (also known as *Lr46*). Their analysis of the results suggested that the *QYr.ucw-1BL* region and *Lr46*/*Yr29* represent the same gene. The researchers highlighted the need to examine the expression of the trait in different genotypes with and without pathogen inoculation to determine whether there are differences in expression levels between candidate genes for *Lr46*/*Yr29*. Thus, we selected four candidate genes for *Lr46*: TraesCS1B02G454200.1 (*Lr46-Glu2*), TraesCS1B02G454000.2 (*Lr46-RLK1*), TraesCS1B02G454100.1 (*Lr46-RLK2*), and TraesCS1B02G454400.1 (*Lr46-RLK3*).

Breeders aim to pyramidise resistance genes within the same cultivar to increase the effectiveness of resistance. Hybrid breeding is the most effective way to combine dominant resistance genes, but its potential to achieve this is strongly dependent on the frequency of genes showing complete dominance. Hybrid wheat breeding is expected to increase yield and stability and is, therefore, used in wheat-breeding programmes. However, basic information on the genetic architecture of wheat and the degree of dominance of rust-disease resistance is still lacking [2]. In order to develop a sustainable and cost-effective leaf-rust control strategy, a collaborative and multidisciplinary research approach involving multiple stakeholders, such as breeders, farmers, geneticists, pathologists, biotechnologists, and policymakers, needs to be implemented [5].

An important factor essential to knowing and understanding the molecular mechanisms of resistance is the regulation of gene expression. MicroRNAs (miRNAs) are small, non-coding endogenous RNAs of 21 to 24 nucleotides (nt) in length that play a key role in the regulation of eukaryotic organisms by inhibiting gene translation or degrading target mRNAs at the post-transcriptional level. Since 2002, when the first plant miRNAs were discovered in *Arabidopsis*, miRNAs have been shown to be involved in many plant processes such as regulation of biogenesis, signal transduction, phase change, and organ development [1,17,23]. Plant miRNAs are also crucial regulators and a key part of plant defence responses [24].

The aim of this study was to analyse the expression patterns of candidate genes for the *Lr46* gene and the *Lr34* and *Lr67* genes responsible for the differentiated resistance to leaf rust of hybrid forms of the F1 generation resulting from crosses between the Glenlea cultivar and cultivars from Polish breeding companies. Moreover, we analysed the expression patterns of five complementary miRNAs (tae-miR9653b, tae-miR5384-3p, tae-miR9780, tae-miR9775, and tae-miR164) complementary to *Lr34* and candidate genes for *Lr46*.

## 2. Materials and Methods

### 2.1. Plant Material

The plant material used in the study consisted of five hybrid forms of the F1 generation obtained by crossing the reference cultivar Glenlea with selected cultivars from Polish breeding companies. Seeds of the Glenlea (“CItr 17272”) cultivar were obtained from the National Small Grains Collection (Agricultural Research Station in Aberdeen, WA, USA). The hybrid forms of common wheat were obtained from four Polish breeding companies: Glenlea × Itaka (Plant breeding Danko, Poland), Glenlea × Merkawa (Plant Breeding Smolice—IHAR Group, Poland), Glenlea × Aura (Plant Breeding Strzelce—IHAR Group, Poland), Glenlea × Jutrzenka, and Glenlea × Harenda (Plant Breeding Małopolska, Poland).

### 2.2. Plant Growth, Pathogen Inoculation, and Leaf Sample Collection

The experiment was conducted in a growth chamber under specified conditions. The temperature was initially set at 18 °C during the day and 16 °C at night. In addition, the emission spectrum of the light source was fixed with a photon flux of 572 μE. One month after setting up the experiment, the temperature was raised to 20 °C and 17 °C during the day and night, respectively. Plants were inoculated at the adult plant stage by spraying a suspension of uredospores, a mixture of *P. triticina* (water with 0.75% Tween 20 reagent) at a concentration of approximately 5 × 10^5^ spores mL^−1^. The mixture was prepared immediately before inoculation. The plant inoculation material was a mixture of four *P. triticina* isolates. Fungus spores were collected from infected field experiments located in various parts of Poland.

### 2.3. Molecular Markers and PCR Reactions

**Figure 1:**
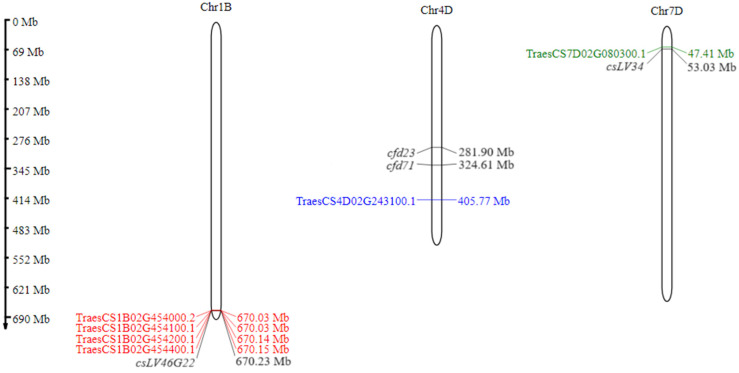
Location of *Lr34*/*Yr18* (TraesCS7D02G080300.1), *Lr67*/*Yr46* (TraesCS4D02G243100.1), and four candidate genes for *Lr46*/*Yr29* (TraesCS1B02G454200.1, TraesCS1B02G454000.2, TraesCS1B02G454100.1, TraesCS1B02G454400.1), on chromosomes 7D, 4D, and 1B, respectively. Candidate genes, including the *QYr.ucw-1BL* region, are highlighted in red. “Ch1B” similarly denotes the chromosome name, and the abbreviation “Mb” next to the scale denotes the megabase unit referring to the length of the chromosome.

The molecular markers *csLV34* [25], *csLV46G22*, *cfd23* [26], and *cfd71* [26] were used to confirm the presence of alleles associated with the *Lr34*/*Yr18*, *Lr46*/*Yr29*, and *Lr67*/*Yr46* genes, respectively (Figure 1). Genomic DNA was isolated from the leaves of 10-day-old seedlings using the GeneMATRIX Plant and Fungi DNA Purification Kit (EURx Ltd., Gdańsk, Poland), according to the procedure provided by the manufacturer. The concentration of isolated DNA was measured using a NanoDrop spectrophotometer and A_260_/A_280_ ratio. Samples were diluted with Elution buffer (EURx Ltd., Gdańsk, Poland) to a uniform concentration of 50 ng µL^−1^.

PCR was performed with FastGene Optima HotStart ReadyMix reagents (NIPPON Genetics Europe GmbH, Düren, Germany), using a Labcycler thermal cycler (SensoQuest GmbH, Göttingen, Germany). The composition of the reaction mixture is given in our previous work [27]. The PCR consisted of initial denaturation at 94 °C for 3 min, followed by 35 cycles (denaturation, 94 °C for 30 s; primer annealing, 60 °C for 30 s; elongation, 72 °C for 90 s), followed by the final extension for 10 min at 72 °C and storage at 4 °C. The primer annealing temperatures of the marker primers were 55 °C for *csLV34* and 60 °C for *cfd23* and *cfd71* markers. In addition, PCR products of the *csLV46G22* marker were digested with the *BspEI* enzyme (Thermo Fisher Scientific, Rockford, IL, USA) at 37 °C for 1 h. PCR products were separated on a 2% High Resolution Plus agarose gel (Bioshop, Canada Inc., Burlington, ON, Canada) in 1 × TBE buffer (Bioshop, Canada Inc., Burlington, ON, Canada), using Midori Green Direct DNA Stain (Nippon Genetics Europe, Germany). Electrophoresis results were visualised in a Molecular Imager Gel Doc™ XR UV system with Bio Image™ software version 5.2.1 (Bio-Rad Laboratories, Inc., Hercules, CA, USA).

### 2.4. Gene Expression Analysis with qRT-PCR

Following recent research [22], ten candidate genes for the *Lr46/Yr29* gene whose sequences were found in the Ensembl Plants database for common wheat (https://plants.ensembl.org/Triticum_aestivum/Info/Index, accessed on 16 April 2023) were selected and downloaded in FASTA format. These sequences were used to design primers for qPCR reactions, using the Primer3Plus tool (https://www.bioinformatics.nl/cgi-bin/primer3plus/primer3plus.cgi, accessed on 16 April 2023). Based on the *T. aestivum* DNA sequence, primers were designed. The designed primers used to analyse the expression of the *Lr34* and *Lr67* genes were described in our previous work [28] and for four candidate genes for *Lr46*. The names and functions of *Lr34*, *Lr67*, and selected candidate genes can be found in Table 1. Isolation of total RNA from leaf tissue samples collected at biological replicates and selected time points (0, 6, 12, 24, and 48 h post-inoculation (hpi)) was performed using the Maxwell RSC Plant RNA Kit isolation (Promega, Madison, WI, USA). The concentration of isolated total RNA was measured using a NanoDrop spectrophotometer and A_260_/A_280_ ratio. First-strand cDNA synthesis was performed using the iScript™ Reverse Transcription Supermix kit for RT-qPCR (Bio-Rad Laboratories, Inc., Hercules, CA, USA), according to the protocol provided by the manufacturer. A temperature gradient PCR was performed for each gene (each primer pair) to determine the annealing temperature. The gradient was set in the temperature range from 50 °C to 58 °C. PCR products were separated on a 2% High Resolution Plus agarose gel (Bioshop, Canada Inc., Burlington, ON, Canada) in 1 × TBE buffer (Bioshop, Canada Inc., Burlington, ON, Canada), using Midori Green Direct DNA Stain (Nippon Genetics Europe, Germany). Electrophoresis results were visualized in a Molecular Imager Gel Doc™ XR UV system with Bio Image™ software version 5.2.1 (Bio-Rad Laboratories, Inc., Hercules, CA, USA). After the specific amplification product was obtained in a 2% agarose gel, the annealing temperature for qRT-PCR was set to 53.5 °C. Two housekeeping genes, *TUBβ* and *ARF*, were selected and qRT-PCR analyses were performed [28].

The qRT-PCR analysis was performed using iTaq Universal SYBR Green Supermix, and CFX Connect Real-Time PCR Detection System (Bio-Rad Laboratories, Inc., Hercules, CA, USA). Each of the qRT-PCR experiments performed consisted of three biological and three technical repeats, the results of which were calculated and averaged. Negative control NTC (No Template Control) was performed during each single expression analysis, applied to a 96-well plate in three technical replicates. The following temperature profile was used for qPCR reactions: initial denaturation for 3 min at 95 °C; then 40 cycles: denaturation for 10 s at 95 °C, annealing of primers for 30 s at 53.5 °C (fluorescence measurement). Melt stage (Melt curve): melting temperature range 65 °C–90 °C; every 5 s the temperature was increased by 0.5 °C and a measurement was taken. The reference genes were tested according to a previously reported protocol [28]. The results of the standard qRT-PCR yield curves (%E) and coefficient of determination (R^2^ values) were included in the calculation of the expression results [28]. The normalised expression was calculated using CFX Maestro software version 4.0 and the Gene Study tool (Bio-Rad Laboratories, Inc., Hercules, CA, USA), which allowed comparison of the expression of all genes for biological repeats of each hybrid form, at selected time points.

### 2.5. Expression Analysis of miRNAs Associated with Lr34 and Candidates Genes for Lr46 Using ddPCR

In this study, the expression of miRNAs was analysed: tae-miR9653b complementary to *Lr34*, three related to the candidate gene *Lr46-Glu2* (tae-miR5384-3p, tae-miR9780, and tae-miR9775), tae-miR9780 complementary to *Lr46-RLK2*, and tae-miR164 complementary to *Lr46-RLK3*. The coding sequences of these candidate target genes, downloaded from the Ensembl Plants database (https://plants.ensembl.org/Triticum_aestivum/Info/Index, accessed on 16 April 2023), were analysed in the psRNATarget database (http://plantgrn.noble.org/psRNATarget/, accessed on 16 April 2023). The sequences of the four miRNAs analysed were found in the miRBase database (https://www.mirbase.org/, accessed on 16 April 2023) and downloaded in FASTA format, and using the IDT—Integrated DNA Technologies website (https://eu.idtdna.com/pages, accessed on 16 April 2023) stem-loop primers were designed for miRNA reverse transcription and ddPCR reactions according to the protocol [29,30,31].

Leaf-tissue samples were collected from each of the five hybrids in three biological replicates. Leaves were placed in tubes and immediately frozen in liquid nitrogen. The collected leaf tissue was stored in a freezer at –80 °C until miRNA isolation was initiated. The mirVana™ miRNA Isolation Kit from ThermoFisher Scientific was used for microRNA isolation, according to the protocol provided by the manufacturer. The primers for the stem-loop RT-PCR were designed based on sequences of complementary miRNA molecules to four genes: the *Lr34* gene and candidate genes for *Lr46* (*Lr46-Glu2*, *Lr46-RLK1*, *Lr46-RLK2*, and *Lr46-RLK3*), according to the protocol [28,29].

### 2.6. Statistical Analysis

Two-way analyses of variance (ANOVA) were carried out to determine the main effects of the hybrid form and time point as well as the hybrid form and time point interactions on the variability of the expression profiles between the genes. In order to compare mean expression at each time point tested after inoculation to expression before inoculation, elemental contrasts were performed, carried out for each hybrid form independently. We referred to the expression values of the miRNAs analysed as ‘relative expression’ for the expression level, as our results represent differences in expression compared to the values before inoculation. The expression of the analysed genes is shown as heatmaps. For visualization of gene expression results, cluster analysis (UPGMA method) with the Euclidean distances was performed. All these analyses were conducted using the GenStat v. 23.1 statistical software package [32].

## 3. Results

### 3.1. Identification of Molecular Markers Associated with the Lr34/Yr18, Lr46/Yr29, and Lr67/Yr46 Locus

The molecular markers used successfully identified selected *Lr* resistance genes in the hybrid forms tested (Table 2). It has been reported in the literature that STS marker *csLV34* is highly reliable for *Lr34* [25,33,34], and *csLV46G22* is a highly reliable and close diagnostic marker for the *Lr46* gene [22,27,35]. In the case of the presence of *Lr34*, we obtained a product of 150 bp. In most of the hybrids analysed, we obtained heterozygotes, only in the case of Harenda × Glenlea was a homozygote (+)obtained (Table 2). In the absence of *Lr34* in the hybrid form, a PCR product of 229 bp (−) would be obtained. Also, the *csLV46G22* marker was identified in all hybrid forms tested. Two flanking SSR molecular markers, *cfd23* and *cfd71*, were used to confirm the presence of the *Lr67* gene (Table 2). In the case of *cfd23*, a PCR product of 211 bp was obtained, while in the case of *cfd71*, it was 214 bp.

### 3.2. Results of the Statistical Analysis

**Figure 2:**
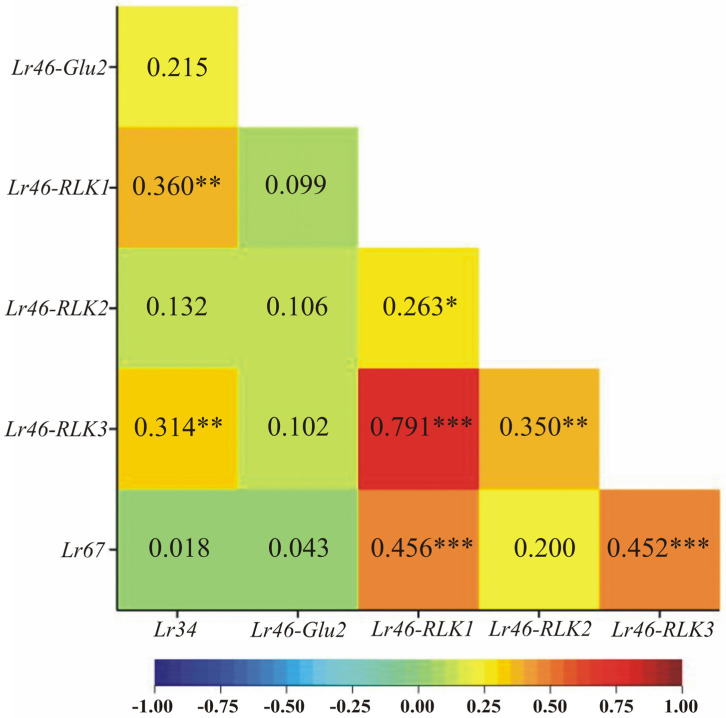
Heatmaps of Pearson’s linear pairwise correlation coefficients between the observed genes *Lr34*, *Lr67*, and the selected candidate genes for *Lr46/Yr29* based on the values of their individual expression profiles. * *p* < 0.05, ** *p* < 0.01, *** *p* < 0.001.

The analysis of variance indicated that the main effects of the hybrid form and time point as well as the hybrid form and time point interaction were significant for the expression profile of all genes. As each gene has a specific expression level, the analysis of variance was performed for each gene separately. An attempt was also made to compare the expression profiles between the genes through Pearson’s linear correlation (Figure 2). As a result of the correlation analysis, it was possible to observe that between *Lr46-RLK1* and *Lr46-RLK3* there is the highest positive correlation (0.791). In contrast, there is almost no correlation between *Lr34* and *Lr67* (0.018) (Figure 2).

The two-way analysis of variance (ANOVA) indicates that the main effect of hybrids was significant for *Lr34*, *Lr46-Glu2*, *Lr46-RLK1*, *Lr46-RLK3*, and *Lr67*. The main effect of time was significant for the expression of all genes except *Lr34*. The main effect of the hybrid × time interaction was significant for the expression of three genes: *Lr46-RLK1*, *Lr46-RLK2*, and *Lr46-RLK3* (Table 3).

### 3.3. Gene Expression Analysis

**Figure 3:**
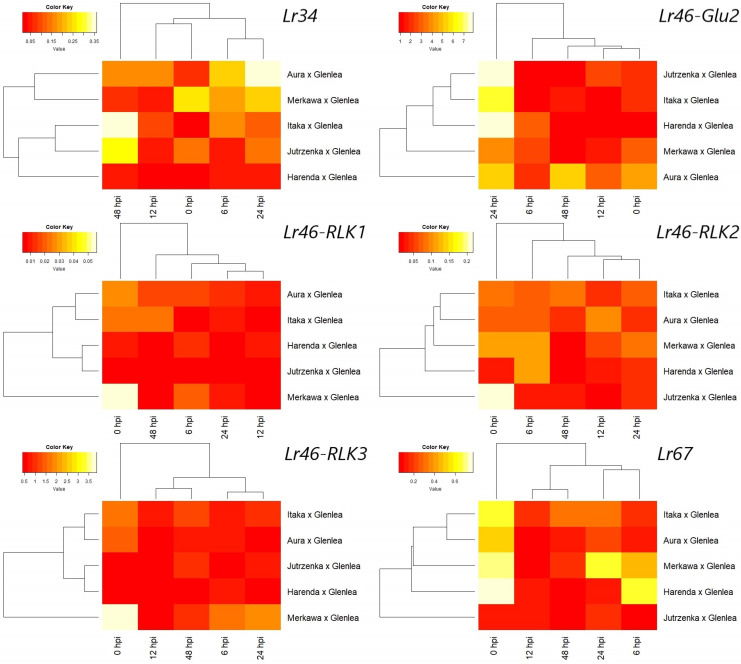
Expression heatmap of the *Lr34*, *Lr67*, and four *Lr46*/*Yr29* candidate genes in leaf tissues under *P. triticina* infection. The evaluation included hybrid forms of common wheat (Harenda × Glenlea, Jutrzenka × Glenlea, Aura × Glenlea, Itaka × Glenlea, and Merkawa × Glenlea) indicated on the vertical axes at different time points (0, 6, 12, 24, and 48 hpi) identified on the horizontal axes. Red indicates lower normalised expression and yellow indicates higher normalised expression.

The qRT-PCR analyses of *Lr34*, *Lr67*, and candidate genes for *Lr46* in all studied wheat hybrids and at different time points are presented as heatmap graphs (Figure 3). Similarly to the previous study [28], the resistance genes *Lr34* and *Lr67* showed a differentiated expression at time points. The *Lr34* gene showed relatively low expression after inoculation of plants by *Pt* fungus. However, compared to our previous work [28], a similarity in the expression profile of *Lr34* in the donor form of Glenlea can be observed in the hybrid forms studied, where the highest expression was observed at 24 and 48 hpi (Figure 3). The exception was the hybrid Harenda × Glenlea, which showed low expression of the *Lr34* gene at all time points (Figure 3). In the Aura × Glenlea hybrid, *Lr34* expression increases intensely immediately after inoculation (6 hpi) and increases again at 24 hpi.

In comparison to *Lr34*, the *Lr67* gene showed a partially different expression pattern, also demonstrated by the analysis of variance (Figure 2 and Figure 3). In the case of the *Lr67* gene, its expression before inoculation is at a remarkably high level (0 hpi); the exception to this trend is the Jutrzenka × Glenlea hybrid, which showed low, constant expression of the *Lr67* gene at all time points (Figure 3). In two wheat hybrids (Harenda × Glenlea and Merkawa × Glenlea), the expression level of this gene decreases slightly after inoculation (6 hpi) and increases again at 24 hpi only for the Merkawa × Glenlea hybrid form. Unfortunately, on normalized expression analysis, *Lr67* gene expression after inoculation did not reach higher values than before inoculation (0 hpi) (Figure 3). This may suggest a weak resistance response to *Pt*.

Among the candidate genes, the least diverse expression profile was observed for the candidate gene *Lr46-Glu2*. In the wheat hybrids, Jutrzenka × Glenlea, Itaka × Glenlea, Harenda × Glenlea, and Merkawa × Glenlea, we observe a strong increase in *Lr46-Glu2* expression at 24 hpi, reaching significantly higher values than before inoculation (Figure 3). The expression level of *Lr46-Glu2* significantly exceeded the pre-inoculation baseline. Interestingly, the expression level of *Lr46-Glu2* at 48 hpi decreased in all tested hybrid forms, but in the hybrid form Aura × Glenlea, it still exceeded the pre-inoculation baseline (Figure 3).

Compared to the candidate gene *Lr46-Glu2*, the genes *Lr46-RLK1*, *Lr46-RLK2*, and *Lr46-RLK3* demonstrate low expression over time, at similar levels in all hybrid forms tested (Figure 3). This may suggest their lower involvement in the defence response after *Pt* inoculation. In the case of the *Lr46-RLK2* gene, expression levels fluctuate over time in all hybrids, eventually reaching lower values than before inoculation (Figure 3). Interestingly, these genes presented very high initial expression (0 hpi) in Merkawa × Glenlea (*Lr46-RLK1*, *Lr46-RLK3)* and Jutrzenka × Glenlea (*Lr46-RLK1)* hybrids.

### 3.4. Expression of miRNAs Complementary to Lr34 and Target Candidate Genes for Lr46

Using stem-loop RT-PCR and ddPCR, we performed expression analysis of miRNA molecules selected from databases as complementary to *Lr34* and four candidate genes for *Lr46*/*Yr29*. We compared the expression results of selected genes with the respective values for the miRNA molecules complementary to them. Contrast analysis was performed for the expression data of miRNAs, and the resulting elemental contrast values for the expression of miRNAs complementary to *Lr34* and candidate genes.

**Figure 4:**
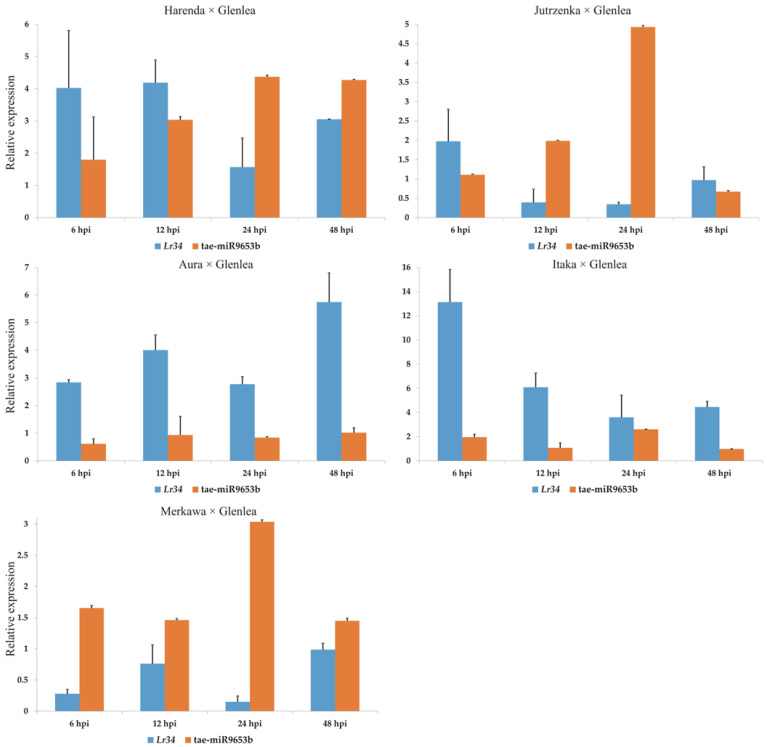
Analysis of complementary tae-miR9653b expression compared to *Lr34* expression patterns during *P. triticina* infection. Numbers 6, 12, 24, and 48 indicate hours post inoculation (hpi). The graphs show the relative expression values compared to the 0 hpi time point. Error bars shown indicate standard error (SE). The expression data shown were obtained from the average of three biological replicates, and each biological replicate had three technical replicates.

Analysis of miRNA using ddPCR revealed a potential role for tae-miR9653b in down-regulation (miRNAs complementary to the *Lr34* gene). The level of sequence-complementary tae-miR9653b fluctuates at observable time points. Eventually, between 24 and 48 hpi, when an increase in *Lr34* gene expression is observed, the level of tae-miR9653b decreases. Such a situation is observed in hybrids Harenda × Glenlea, Jutrzenka × Glenlea, and Merkawa × Glenlea, where the expression levels of *Lr34* and tae-miR9653b genes fluctuate after inoculation. In the case of Jutrzenka × Glenlea and Merkawa × Glenlea, we observed a significant increase in tae-miR9653b expression at 24 hpi, well above the baseline (Figure 4). In the case of Aura × Glenlea and Itaka × Glenlea, we do not observe significant tae-miR9653b expression, which is obtained at consistently low levels at all selected time points (Figure 4).

**Figure 5:**
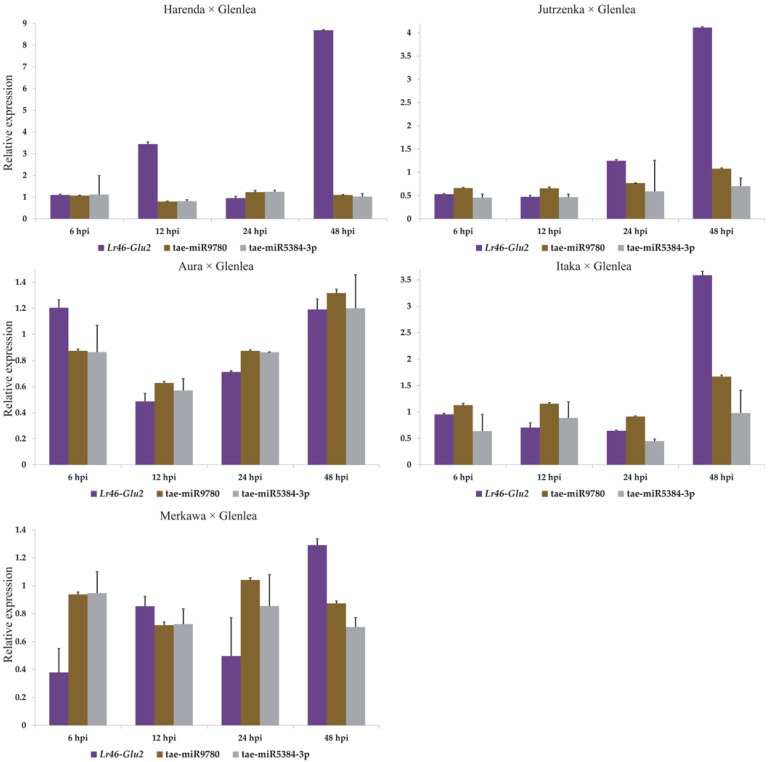
Analysis of complementary tae-miR9780 and tae-miR5384-3p expression compared to *Lr46*-*Glu2* expression patterns during *P. triticina* infection. Numbers 6, 12, 24, and 48 indicate hours post inoculation (hpi). The graphs show the relative expression values compared to the 0 hpi time point. Error bars shown indicate standard error (SE). The expression data shown were obtained from the average of three biological replicates, and each biological replicate had three technical replicates.

According to the database, tae-miR9780 is complementary to two candidate genes, *Lr46-Glu2* (TraesCS1B02G454200.1) and *Lr46-RLK2* (TraesCS1B02G454100.1). Relative to the *Lr46-Glu2* gene, tae-miR9775 is also complementary. Unfortunately, the expression of tae-miR9775 was at a low level and did not allow for confident inference. In the *Lr46-Glu2* gene, we analysed the expression of two miRNAs: tae-miR9780 and tae-miR5384-3p. The expression of tae-miR9780 in the majority of the tested wheat hybrids was at a constant level, both before and after inoculation. Analysing the complementary to this gene tae-miR5384-3p, it was found that its expression level after inoculation in four hybrids fluctuated eventually taking values lower or equal to the initial values before inoculation. Therefore, this can suggest that tae-miR5384-3p did not specifically repress the *Lr46-Glu2* gene, as gene expression increased after inoculation. Of all the hybrids analysed, only Aura × Glenlea showed a significant increase in tae-miR5384-3p expression at 48 hpi, compared to pre-inoculation levels. For the other wheat hybrids, a lower copy number of this miRNA was recorded at 48 hpi than before inoculation by *Pt* (Figure 5).

**Figure 6:**
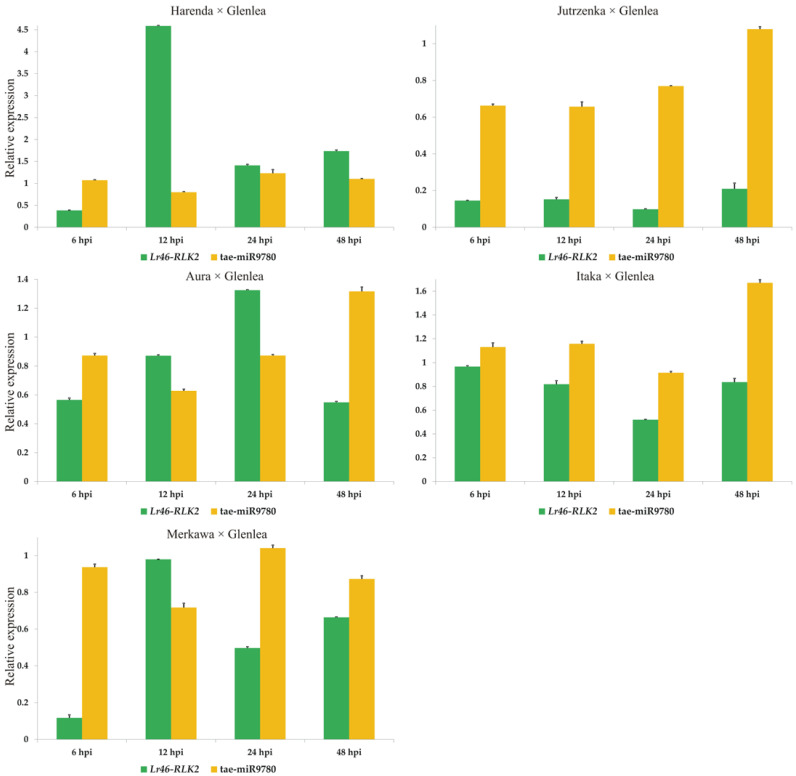
Analysis of complementary tae-miR9780 expression compared to *Lr46*-*RLK2* expression patterns during *P. triticina* infection. Numbers 6, 12, 24, and 48 indicate hours post inoculation (hpi). The graphs show the relative expression values compared to the 0 hpi time point. Error bars shown indicate standard error (SE). The expression data shown were obtained from the average of three biological replicates, and each biological replicate had three technical replicates.

Comparing the expression of tae-miR9780 molecules with the expression profile of *Lr46-RLK2*, we observed variability among the hybrids tested (Figure 6). In each hybrid form (except Harenda x Glenlea), we observed the highest expression of tae-miR9780 at 48 hpi. The fluctuation in tae-miR9780 expression may suggest its possible role in resistance response to biotic stresses.

**Figure 7:**
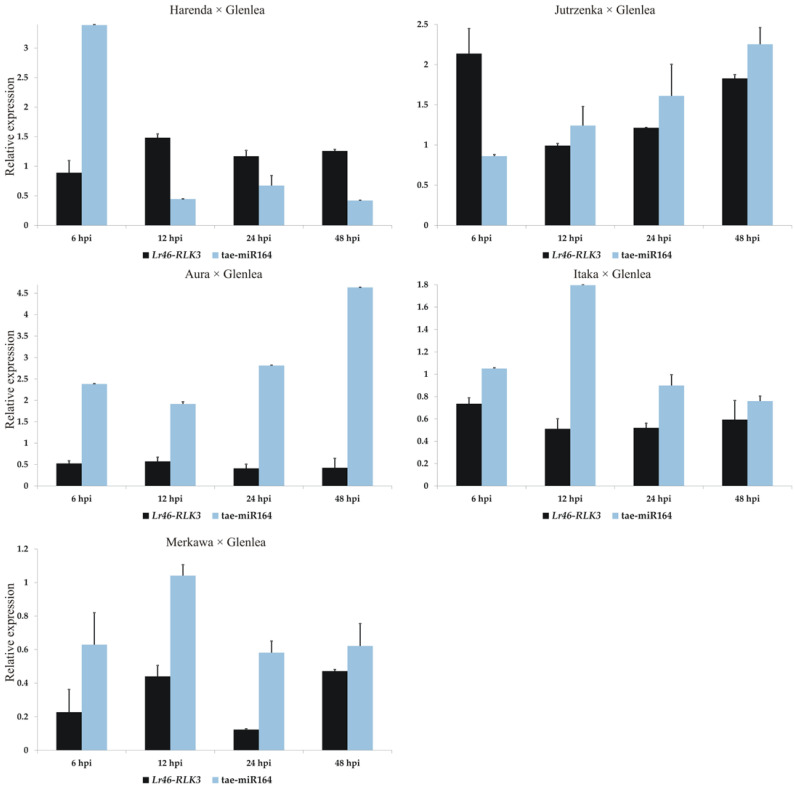
Analysis of complementary tae-miR164 expression compared to *Lr46*-*RLK3* expression patterns during *P. triticina* infection. Numbers 6, 12, 24, and 48 indicate hours after inoculation (hpi). The graphs show the relative expression values compared to the 0 hpi time point. Error bars shown indicate standard error (SE). The expression data shown were obtained from the average of three biological replicates, and each biological replicate had three technical replicates.

In contrast, tae-miR164, which is complementary to the candidate gene *RLK3* (TraesCS1B02G454400.1), showed differential expression at pre- and post-inoculation. In the case of the two hybrid forms, Jutrzenka × Glenlea and Aura × Glenlea, we observed a significant increase in the tae-miR164 expression, relative to the pre-inoculation value. For the other hybrids, tae-miR164 expression fluctuated between 6 hpi and 48 hpi after inoculation eventually reaching a lower value than before inoculation (Figure 7). In the case of this candidate gene, there were also no significant correlations between its expression and that of tae-miR164, which is complementary to its sequence.

## 4. Discussion

According to our research hypothesis, only one gene is responsible for the desired *Pt* type APR resistance. Nevertheless, it is possible that there is a cluster of closely related resistance genes, each of which is effective against different pathogens. Although cloning of these genes would be necessary to provide a definitive test of this hypothesis, examining the ability of the *QYr.ucw-1BL* region to produce APR resistance against leaf rust, stem rust, and powdery mildew pathogens may provide valuable information to confirm the above hypothesis. In our previous study, we were able to analyse the expression profiles for nine of the ten candidate genes included in the *Lr46/Yr29* locus at five time points (0, 6, 12, 24, and 48 hpi): TraesCS1B02G453900.1, TraesCS1B02G454200.1, TraesCS1B02G454500.1, TraesCS1B02G454000.2, TraesCS1B02G454100.1, TraesCS1B02G454400.1, TraesCS1B02G454600.1, TraesCS1B02G453700.1, and TraesCS1B02G455000.1. In this study, we showed that of all the genes analysed, the candidate gene *Lr46*-*Glu2* showed the highest expression level after inoculation by *P. triticina*. This may indicate its important role in resistance processes. Another gene with lower but significant expression was another *Lr46* candidate gene, *Lr46*-*RLK3*. The results indicate that the candidate gene *Lr46*-*Glu2* may be involved in the resistance response of the plants. After inoculation by *Pt* fungus, for all hybrid combinations 24 hpi after inoculation, its expression level increases intensely reaching higher values than before inoculation. In our previous study, we observed the same trend in the *Lr46-Glu2* expression profile at the selected time points after inoculation by *Pt*. The *Lr46-Glu2* analysis included five reference-resistant wheat cultivars, where its highest expression was observed at 24 and 48 hpi. The expression of this gene was also not potentially down-regulated by the complementary to its sequence tae-miR5384-3p.

Compared to the *Lr46*-*Glu2* candidate gene, the *Lr46*-*RLK3* gene appears to be less involved in the defence response after *P. triticina* infection, since in up to three hybrids (Itaka × Glenlea, Aura × Glenlea, and Merkawa × Glenlea), its expression levels fluctuated over time, eventually taking on lower values than before inoculation. Among the selected candidate genes in the work of Cobo et al. [22], three candidate genes (TraesCS1B02G453900.1, TraesCS1B02G454200.1, and TraesCS1B02G454500.1) are *ENDO-1,3-BETA-GLUCAN GLUCOSIDASE*, enzymes belonging to the PR2 class, involved in the response of wheat to biotic stresses. According to the literature, these are proteins involved in plant defence mechanisms through hydrolysis of the fungal cell wall. In a recent study, an increase in endo-1,3-beta-glucosidase glucan was observed during drought stress [36]. β-1,3-glucanases belong to the PR-2 family of plant pathogenesis-related proteins (PRs). They are enzymes that are abundant in plants and have numerous functions related to cell division, are involved in transport through plasmodesmata and in the resistance response to stresses [37].

Many receptor-like kinase (RLK) gene families can be found in plants. In the work of Cobo et al. [22], four of the candidate genes are RLK genes (TraesCS1B02G454000.2, TraesCS1B02G454100.1, TraesCS1B02G454400.1, and TraesCS1B02G454600.1), which encode proteins important for recognising extracellular signals and initiating intracellular signalling cascades in response to these stimuli. RLKs in this region encode proteins with two extracellular domains that are characteristic of a subgroup of cysteine-rich receptor kinases [22,38,39]. It has been proposed that members of this sub-group may be involved in redox signalling [38]. RLKs are membrane proteins located in the extracellular domain of the receptor and are involved in both biotic and abiotic stress responses. The extracellular ligand-binding domain, the transmembrane domain, and the intracellular protein kinase domain are typical components of RLKs. The extracellular domain, which is specific to individual RLKs, binds a particular ligand and allows RLKs to respond to different types of signals. Among the receptor-like kinases (RLKs), variants such as leucine-rich repeats (LRR), lectin (Lec), lysine motif (LysM), or wall-associated kinases (WAK) are distinguished [39,40]. receptor-like protein kinases (rlk) make the largest known family of protein kinases that are important in plant response to pathogen infection. Gu et al. [39] discovered a new cysteine-rich RLK gene, *TaCRK2*, which positively regulates resistance to infection caused by *Pt* in wheat. In a recent study by Hajiahmadi et al. [41], researchers investigated the expression of differentially expressed genes induced by biotic stress induced by *P. triticina*, which was characterised in an isogenic line carrying the *Lr57* gene. The highest number of transcripts undergoing expression was detected in 12 hpi. Interestingly, among these transcripts, a cysteine-rich receptor-like protein kinase was expressed only in the resistance genotype at 12 hpi.

In this study, we also analysed expression patterns of tae-miR5384-3p. We observed that the tae-miR5384-3p expression level after inoculation fluctuated, eventually taking values lower or equal to the initial values before inoculation. Therefore, this can suggest that tae-miR5384-3p did not specifically repress the *Lr46-Glu2* gene, as gene expression increased after inoculation. However, one of the target functions of tae-miR5384-3p may be to inhibit a number of genes in the TaFAB2 subfamily that are associated with the formation of unsaturated fatty acids, which play an important role in plant development and in response to biotic and abiotic stresses [42]. In contrast, tae-miR5384-3p showed a very high increase in expression level after treating wheat seedlings with a chitosan suspension [43]. The type of fluctuating relative expression profiles is commonly observed for small RNA sequencing or qRT-PCR-based miRNA expression analysis under different biotic and abiotic stress conditions [44]. Such a phenomenon may relate to changing defence mechanisms in the early and late stages of the plant response [44].

In comparison to model plants such as rice and *Arabidopsis*, research into the role and function of miRNAs in wheat has been significantly delayed. The challenging aspect is the common wheat genome, which is huge (approximately 16 Gbp) and complex containing repetitive sequences, including retrotransposons, that account for as much as about 85% of the genome [24,45]. Plant tolerance to abiotic and biotic stresses are only a few of the biological or agronomic traits that can be improved with new biotechnology tools based on miRNA-encoding *MIR* genes. A more profound understanding of the regulation and expression profile of *MIR* genes related to intrinsic traits of RNA interference (RNAi) mechanisms, tolerance to abiotic or biotic stresses, and exploiting the advantages offered by new biotechnology tools can lead to practical biotechnology applications to improve agronomic traits in many crops worldwide. Furthermore, with this knowledge, new biotechnological products can be created with greater practicality, reduced generation time, and lower costs [46].

The miR164 family is one of the most conserved groups of miRNAs in plants [24]. One miRNA molecule can control multiple target genes, similar to plant transcription factors. Previous studies have shown that miR164 targets plant-specific NAC transcription factors [1]. The majority of NACs play an essential role in the regulation of plant development and in the response to abiotic and biotic stresses. The functions of miR164 have been identified in plant responses to biotic stresses. The main function attributed to miR164 is to regulate transcript levels of relevant genes [47,48,49]. This miRNA also targets the selected candidate gene *Lr46*-*RLK3*. In our study, in the majority of tested wheat cultivars, when candidate gene expression increased, tae-miR164 was down-regulated, and vice versa: when miRNA expression increased, the candidate gene expression values were down-regulated. The decrease in tae-miR164 may be related to the activation of *Lr46*-*RLK3* gene-dependent immune mechanisms, observed as a rapid gene response to the pathogen. Moreover, we observed similar interactions in our previous work on tae-miR164 [34]. Recent studies on miRNAs associated with the endoplasmic reticulum (ER) stress response have shown that tae-miR164 plays a negative regulatory role in wheat in response to drought, salt, and heat stress. The tae-miR164 molecule, together with tae-miR2916 and tae-miR396e-5p, has been identified as an ER stress-responsive miRNA [17].

## 5. Conclusions

In this study, we analyse the expression profiles of *Lr34*, *Lr67*, four candidate genes located at the *Lr46* locus, and five complementary miRNAs (tae-miR9653b, tae-miR5384-3p, tae-miR9780, tae-miR9775, and tae-miR164), in response to *Pt* infection. Sustained host-plant resistance to leaf rust is one of the most important traits that breeding programmes should invest in, allowing for reduced fungicide use and promoting greater stability and sustainability of yield levels. The effect of gene combinations, the presence of unidentified genes, and different expression levels of resistance genes as well as other biotic and abiotic factors can cause different responses of cultivars carrying the same resistance genes to *P. triticina*. Sources of race-specific and non-race-specific genes can be used to be pyramided with other effective *Lr* genes. The use of highly advanced and high-throughput tools such as field pathogenomics, transgenetics, and genome editing to study both host and pathogen organisms will be helpful in achieving broad-spectrum and durable resistance to leaf rust in common wheat.

In our study, we analysed the genes and miRNAs expression pathways in hybrid forms: Harenda × Glenlea, Jutrzenka × Glenlea, Aura × Glenlea, Itaka × Glenlea, and Merkawa × Glenlea. The *Lr34* gene, as in our previous study, showed relatively low expression at the time points studied. However, it is possible to observe that *Lr34* expression at 24 and 48 hpi increases, and tae-miR9653b expression decreases. Therefore, it can be concluded that tae-miR9653b may be involved in the control of the defence response. Among the candidate genes for *Lr46*, the least diverse expression profile was observed for the candidate gene *Lr46-Glu2*, with an increase in its expression at 24 hpi, which may suggest activation of the defence response in tested hybrid forms. The expression level of *Lr46-Glu2* significantly exceeded the pre-inoculation baseline in all tested wheat hybrids.

Our research highlights the need to understand the molecular basis of plant-pathogen interactions, and this will enable the development of new strategies for *Pt* resistance. To determine the allelic variation of the *Lr46* locus gene(s), cloning and functional analysis of the gene(s) is essential. This achievement would allow the development of more specific molecular markers for *Lr46*, which would facilitate line selection in wheat resistance breeding.

## Figures and Tables

**Table 1:** Presentation of *Lr34/Yr18*, *Lr67/Yr46*, and four selected genes located in the candidate region for *Lr46*/*Yr29* gene, their localization, and predicted function, involving the *QYr.ucw-1BL* region.

Gene ID	Gene Name	Gene Predicted Function	Physical Region (Mb)
TraesCS7D02G080300.1	*Lr34*	ABC transporter	7D: 47,412,062–47,424,490
TraesCS4D02G243100.1	*Lr67*	hexose transporter	4D: 405,770,757–405,775,531
TraesCS1B02G454200.1	*Lr46-Glu2*	gucan endo-1,3-β-glucosidase	1B: 670,142,374–670,144,629
TraesCS1B02G454000.2	*Lr46-RLK1*	RLK (receptor-like kinase)	1B: 670,025,362–670,028,705
TraesCS1B02G454100.1	*Lr46-RLK2*	RLK (receptor-like kinase)	1B: 670,034,245–670,037,207
TraesCS1B02G454400.1	*Lr46-RLK3*	RLK (receptor-like kinase)	1B: 670,152,915–670,155,867

**Table 2:** Summary of the presence of the molecular markers *csLV34*, *csLV46G22*, *cfd23*, and *cfd71* in tested common wheat hybrids.

No.	Wheat Hybrid	*Lr34* (*csLV34*)	*Lr46* (*csLV46G22*)	*Lr67* (*cfd23*)	*Lr67* (*cfd71*)
1	Harenda × Glenlea	+	+	+	+
2	Jutrzenka × Glenlea	H	+	+	+
3	Aura × Glenlea	H	+	+	+
4	Itaka × Glenlea	H	+	+	+
5	Merkawa × Glenlea	H	+	+	+

+, presence; H, heterozygous.

**Table 3:** Mean squares from two-way analysis of variance for expression profiles of studied genes in hybrid forms of wheat.

Source of Variation	Hybrid	Time	Hybrid × Time Interaction	Residual
Degrees of freedom	4	4	16	50
*Lr34*	0.055 *	0.036	0.025	0.015
*Lr46-Glu2*	5.671	52.391 ***	5.630	4.194
*Lr46-RLK1*	0.00046 ***	0.00066 ***	0.00025 ***	0.00003
*Lr46-RLK2*	0.0023	0.0107 *	0.0057 *	0.0029
*Lr46-RLK3*	3.625 ***	1.945 ***	1.016 ***	0.122
*Lr67*	0.207 *	0.439 ***	0.083	0.066

* *p* < 0.05; *** *p* < 0.001.

## Data Availability

The data presented in this study are available on request from the corresponding author.
